# The tonic immobility test: Do wild and captive golden mantella frogs (*Mantella aurantiaca*) have the same response?

**DOI:** 10.1371/journal.pone.0181972

**Published:** 2017-07-21

**Authors:** Luiza Figueiredo Passos, Gerardo Garcia, Robert John Young

**Affiliations:** 1 School of Environment and Life Sciences, Peel Building, University of Salford Manchester, Salford, United Kingdom; 2 Chester Zoo, Cedar House, Upton by Chester, Chester, United Kingdom; University of Sao Paulo, BRAZIL

## Abstract

Adaptations to captivity that reduce fitness are one of many reasons, which explain the low success rate of reintroductions. One way of testing this hypothesis is to compare an important behavioural response in captive and wild members of the same species. Thanatosis, is an anti-predator strategy that reduces the risk of death from predation, which is a common behavioral response in frogs. The study subjects for this investigation were captive and wild populations of *Mantella aurantiaca*. Thanatosis reaction was measured using the Tonic Immobility (TI) test, a method that consists of placing a frog on its back, restraining it in this position for a short period of time and then releasing it and measuring how much time was spent feigning death. To understand the pattern of reaction time, morphometric data were also collected as body condition can affect the duration of thanatosis. The significantly different TI times found in this study, one captive population with shorter responses, were principally an effect of body condition rather than being a result of rearing environment. However, this does not mean that we can always dismiss the importance of rearing environment in terms of behavioural skills expressed.

## Introduction

Considerable difficulty has been encountered in successfully reintroducing endangered species into their natural habitats, and adaptations to captivity that reduce fitness in the wild (e.g. lack of predator recognition and appropriate response) are one of several reasons for this low success rate [1]. If captive animals are to be released into the wild, these issues should be addressed [2]. Evaluating the behavioural skills of captive bred animals could allow the selection of appropriate individuals and lead to improvements in the success rates of reintroduction programs [3]. This has been showed for different species such as black-footed ferrets (*Mustela nigripes*) [3], Caribbean rock iguanas (*Cyclura sp*.) [4] and different fish species [5].

One of the most important responses to preserve in captive populations destined for reintroduction is the ability to detect and respond appropriately to natural predators [4,6]. It is known that captivity can cause animals to: lose natural responses, have insufficient fear of humans, and express abnormal behaviour [5,7,8]. These can limit the success of subsequent reintroduction attempts [5,7,8]. An example is the first attempts to release golden lions tamarins *(Leontopithecus rosalia)*, that failed due the lack of behavioral skills possessed by captive reared individuals [3]. Captive environments are often highly predictable and without threatening stimuli, this could lead to important anti-predator responses being weakened or even disappearing during generations of captive breeding [5,6,9].

Tonic immobility (TI), or thanatosis, is behavioural motor inhibition and reduced responsiveness to external stimulation induced by physical restraint [10]. The TI response is considered as an adaptive behavioural anti-predator strategy, reducing the threat of death from predation and, thereby, increasing the chances of survival [11]. While displaying thanatosis an animal adopts a posture that gives it the appearance of being dead with which it may inhibit or divert the attack of a potential predator [11]. Toxic animals, such as golden mantella frogs, display conspicuous body coloration, and their immobile posture would often enhance the effectiveness of aposematism [12]. Tonic immobility could induce the predator to loosen its hold on the prey, thereby providing a chance of escape [11,13].

Tonic Immobility has been documented as a behaviour expressed by a wide variety of species including mammals, insects, reptiles, birds, fish and amphibians [10,11,13,14,15,16]. This response seems specific to threatening situations; the more intense the stimulus is, the longer the TI response is [11]. It is known that different factors can influence thanatosis duration such as stress levels [17], welfare status [13], stimulus intensity [18], predation pressure [19] and environmental disturbances [20] amongst others. Studies with frog species have demonstrated that stressful stimuli such as loud noises (*Rana pipiens*, [20]), extreme temperatures (*Rana temporaria*, [21]) or the sight of predators (*Platymantis vitiana*, [18]) can affect TI response duration of captive animals.

It is crucial to conserve the behavioural integrity of captive wildlife, particularly if animals are to be used for conservation efforts including reintroductions [22,23]. Therefore, investigations as to whether captive breeding centres are providing the stimuli to allow species to fully develop their behavioral repertoire are crucial [23]. The aim of this study was to compare tonic immobility responses of wild and captive golden mantella frogs (*Mantella aurantiaca)*, thereby assessing the effects of captivity on this survival strategy. As death feigning is a natural defensive response [11, 14, 18] it was predicted that wild frogs will have a longer TI response since these individuals are expected to be more experienced in expressing defensive behaviours due to the threats in their habitat. Captive bred animals can be naive to the threat of predation and, therefore, might be unable to generate adequate physiological and behavioural responses to a threatening stimulus [18]. Tonic immobility is also associated with fear [18], since captive frogs are also habituated to handling and human interaction (e.g. during cleaning and feeding routines): a human interaction should not trigger such a fear response [24].

## Methodology

### Ethical approval

All the research reported in this study was approved by the Ethics Commission of Chester Zoo, UK and it conforms to all regulations and laws in all relevant countries in relation to care of experimental animal subjects. Furthermore we can confirm, from our post-experimental monitoring, that no animals suffered any injuries, became ill or had their survivorship negatively affected as a result of this study.

### Study subjects

The model species for this study was the golden mantella frog (*M*. *aurantiaca)*. It is a species classified as critically endangered by the IUCN [25] and is endemic to the Moramanga district, in the Region of Alaotra-Mangoro, Madagascar. It is well known due to its aposematic orange-red colouration and presence in the international pet trade [25]. Potential predators for the species would be reptile species such as *Zonosaurus madagascariensis* and *Tamnosophis lateralis* [26]. Its distribution is restricted to a fragment of humid forest around seasonally flooded ponds surrounded by degraded land [25]. A significant proportion of its population is located inside or near the area of the Ambatovy mine [27]. Following a conservation needs assessment, the Amphibian Ark prioritised *M*. *aurantiaca* as a species in need of *ex situ* assistance to safeguard its survival [27,28,29].

### Study sites

#### Mangabe area

Mangabe rainforest is a site of international biodiversity importance, being home to almost half of the world's breeding ponds for the golden mantella frog according to recent studies on high conservation priority sites for mantella frogs. Mangabe forest, or the 'blue forest', covers approximately 40,000 ha in eastern Madagascar and is divided between two administrative districts, Moramanga in the north and Anosibe An'ala to the south. Data sampling for this study was done in the Moramanga region. The data from wild frogs (N = 90) at Mangabe were obtained during October 2014 and again in February 2015.

#### Ambatovy mining site

Ambatovy’s mine is located within a species-rich region of Madagascar at the southern end of the remaining Eastern Forest Corridor in the Moramanga region. As part of the Environmental Management Plan, there is a Conservation Zone of native forest maintained by the mining company. Pre-clearance species inventories and translocation of live animals to conservation forest refuge areas called the Receptor Ponds were carried by Madagasikara Voakajy, a local NGO involved in the conservation of golden mantellas. During this study, animals from the Conservation Zone and animals that were translocated to Receptor ponds were sampled. Ambatovy population (N = 30) was sampled in March 2016.

#### Chester Zoo, UK

Chester Zoo is actively involved in the conservation of the golden mantella frogs in Madagascar. The zoo currently maintains two *ex situ* groups of *M*. *aurantiaca*, one is on public display at the Zoo’s Tropical Realm exhibit and a second group is kept off show in a biosecurity container specifically for conservation-related research. Frogs are kept in naturalistic tanks with different live species of plants, moss for substrate, water, hiding places under rocks, UV light and heaters to mimic the natural conditions found in Madagascar. Animals are fed different live invertebrates with diet supplementation. The Chester Zoo population (N = 30) was sampled in March 2016.

#### Mitsinjo Association Captive Breeding Centre

Mitsinjo Association is a community-run conservation organization. This is Madagascar’s first biosecure facility to safeguard amphibians from extinction, and currently maintains a genetically viable population of the golden mantella frog taken from the Ambatovy mining site (i.e., genetic founders). The offspring (F1) of these individuals are intended for reintroductions at artificially created breeding and natural ponds. Animals are kept in tanks with aquarium gravel as substrate, a plant pot, water, coconut shells for hiding. No UV light was supplied. Animals were fed a variety of live invertebrates, but no food supplementation is given. During this project, only data from the founders’ offspring (F1) were collected. The data from the captive frogs from the Mitsinjo captive breeding centre (N = 20) were obtained in February 2015.

### TI test

Thanatosis reaction was measured using the Tonic Immobility (TI) test, a standardised method that consistently and reliably induces TI [10,13]. Frogs were caught and immediately subjected to the TI test (within 3 s). Each individual was placed on its back in the palm of the experimenter’s hand and restrained in that position for 10 s using gentle pressure on its belly from the experimenter’s thumb, and then released. If a frog moved 3 s after release, then it was considered that TI had not been induced. In this case, the restraint was repeated up to three times. If TI was not induced after 3 attempts, a score of 0 s was given. Conversely, if frogs did not show any movement after 5 min, the test was terminated and a maximum score of 300 s was given for tonic immobility duration. Animals were always handled by the same researcher. Tonic immobility can be affected by ambient temperature [15,21], Chester Zoo facilities are kept in a temperature controlled environment to mimic Madagascar climate conditions. Mitsinjo facilities’ temperature is allowed to fluctuate with the climate outside since the captive population was maintained within the native range of the species [25]. For this reason temperature was not used as a possible source of variation (i.e. factor).

### Body condition index

Body condition index (BCI) was assessed using the Scaled Mass Index proposed by Peig and Green [30]. This method is independent of size and can be used for comparison between different populations; these characteristics potentially make it superior to the traditional residual indices and, reportedly it has worked well in amphibian studies [31,32]. The scaled mass index of condition (M_i_) was calculated as follows:

$$M_{i}=M*{\left[\frac{{SVL}_{o}}{SVL}\right]}^{bSMA}$$

where M and SVL are the mass and Snout-vent length of the individual, SVL_0_ is the arithmetic mean SVL of the population, and bSMA is the standardized major axis slope from the regression of ln M on ln SVL for the population [30]. Each individual was measured (±0.01mm) for SVL using a digital calliper (Lujii 150mm, Omiky) and body mass was obtained using a precision scale (accurate to 0.01g, Smart Weigh ACC200 AccuStar).

### Data analysis

Data were confirmed to have a normal distribution using the Shapiro-Wilk normality test. There were no statistical differences between BCI and TI responses between the two sample periods in Mangabe, and between the two populations from Chester Zoo, for this reason, data were analysed together. TI responses and BCI were compared using ANOVA tests. A Pearson correlation was used to analyse BCI and TI responses. Statistical analyses were done using R Studio [33].

## Results

There was no significant difference in TI responses among groups (wild and captive) (F = 1.901, df = 1, p = 0.17), but there was a significant difference between populations (F = 12.23, df = 4, p<0.001). The Tukey *post-hoc* analyses showed that the golden mantella frog population kept at Mitsinjo Breeding Centre had a significantly (p<0.01) shorter duration TI response when compared to all other groups (Table 1) and no other significant differences were detected.

**Table 1:** Tonic immobility test results for different wild and captive populations of golden mantella frogs.

Population	Group	N	Max (secs)	Min (secs)	Mean (secs)	St. Dev (secs)
Mangabe	Wild	90	180	0	78.54	47.40
Ambatovy—Receptor	Wild	30	147	0	81.00	67.00
Ambatovy -Conservation	Wild	30	180	0	71.31	59.06
Mitsinjo Breeding Centre	Captive	20	40	0	10.05	13.72
Chester Zoo	Captive	30	136	30	83.63	29.99

After obtaining a body condition index for all individuals (Table 2), groups (wild x captive) were compared using a one-way ANOVA test (F = 8.278, df = 1, p = ns). The test showed that there was no significant difference between groups.

**Table 2:** Body condition index score results for different wild and captive populations of golden mantella frogs.

Population	Group	N	Max	Min	Mean	St. deviation
Mangabe	Wild	90	1.54	0.42	0.89	0.16
Ambatovy—Receptor	Wild	30	2.29	0.56	0.88	0.40
Ambatovy -Conservation	Wild	30	1.01	0.49	0.87	0.11
Mitsinjo Breeding Centre	Captive	20	1.28	0.39	0.67	0.19
Chester Zoo	Captive	30	1.12	0.40	0.91	0.32

There was no significant difference on the body condition index between groups (wild and captive (F = 0.569, df = 1, p = 0.45) and a significant difference between populations (F = 9.289, df = 4, p<0.001). The Tukey *post-hoc* analyses confirmed that animals from Mitsinjo were significantly different from all other groups with a much lower body condition.

**Fig 1:**
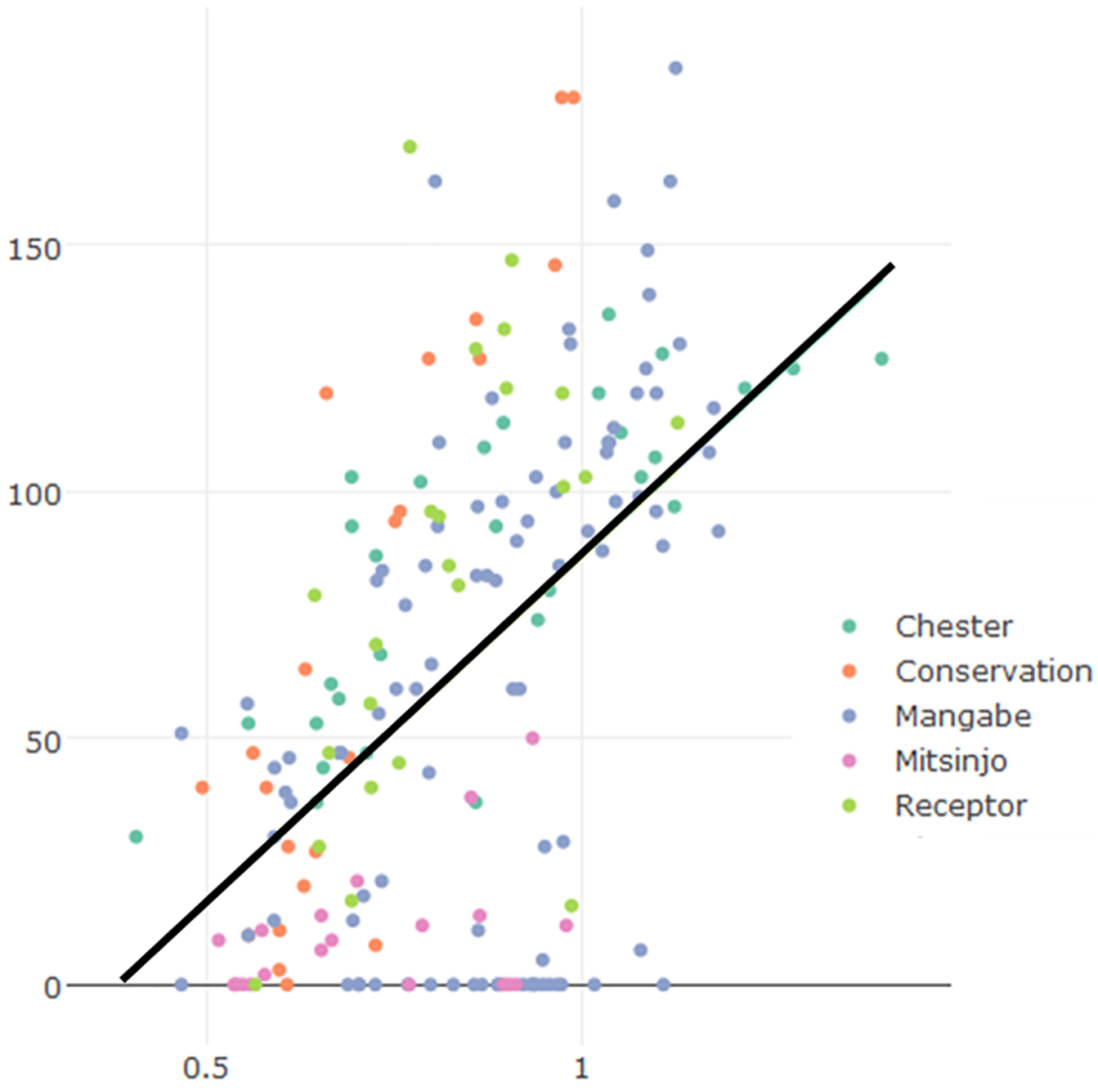
Scatter plot of body condition index (BCI) and tonic immobility (TI) response (s) of different populations of golden mantella frogs.

A significant positive correlation was found between TI responses and body condition index scores when all data were compared using a Pearson correlation test (r = 0.02, N = 200, p<0.05.; observation: 4 outliers removed (r = 0.33, N = 196, p<0.001), which had very large standardised residuals) and when each population was analysed separately (Table 3, Fig 1). Animals with better body condition had longer responses.

**Table 3:** Pearson correlation results for relationship between tonic immobility response (duration) and body condition index for different golden mantella frog populations.

Population	r	N	p-Value
Mangabe	0.06	90	<0.05
Ambatovy—Receptor	0.07	29	<0.05
Ambatovy -Conservation	0.15	29	<0.01
Mitsinjo Breeding Centre	0.06	19	<0.05
Chester Zoo	0.04	29	<0.01

## Discussion

In this study we showed that wild populations of golden mantella frogs and those kept at Chester Zoo had similar TI response durations, whereas animals kept at Mitsinjo breeding centre had a significantly shorter TI duration. These results suggest that captivity is not the only factor involved in the shorter durations observed in one of the captive colonies. Animals from Chester Zoo, which have been in captivity for many more generations, still presented the same response as the wild populations. On the other hand, frogs kept at Mitsinjo breeding centre after the first generation in captivity presented a shorter response when compared to wild animals. This is true even when compared to the wild population from where their parental generation were collected, which also discounts the results being due to some natural variation between populations.

During this study, there was also a significant difference in the body condition of animals between the populations. Body condition is a valuable index that can be assessed using reliable, non-invasive techniques, and it can identify the health condition of a population before any deleterious effects can be observed [31]. The data collected from wild and captive *M*. *aurantiaca* showed that the individuals kept at the Mitsinjo breeding centre had a much lower body condition index than any other group. Again, this cannot be generalized as a consequence of captivity, since frogs from Chester Zoo present no statistical difference on BCI when compared to the wild populations. This result could be used to infer that animals at Mitsinjo are not in ideal health condition when compared with other analysed populations.

Lower body condition could be a result of different factors such as diet, reproductive stage and age [34]. Both captive colonies receive a diet of variety of live invertebrates, but Chester Zoo’s colony also received a diet supplementation. There is a lack of knowledge concerning the nutritional necessities and absorption efficiency of amphibians; however, studies have demonstrated that diet supplementation can have a positive impact on frog body condition and general health [35]. This lack of vitamin and mineral supplementation could be causing frogs from Mitsinjo to have a lower body condition.

There is also a reported relationship between weight-loss and stress in captive individuals [17,34]. Captivity can present many sources of stress, possibly the greatest stressors are those over which the animal has no control and from which they cannot escape, such as a poor diet, inadequate habitat and restricted movement [17]. Chronic stress may be indicated by a wide range of physiological responses including inhibited growth rate [36,37], reduced body weight [38,39], and reduced food intake [40]. Persistent exposure to continuous stressors can have many deleterious consequences for captive animals putting at risk the long-term health of captive animals [23,36,41,42,43,44]. Environmental factors, such as providing the correct UV light standards, could be involved in maintaining the healthy state of frogs kept in captivity [32,45,46] The lack of UV light provision for the Mitsinjo colony could, also, be involved at the low body condition.

The positive correlation between TI response and BCI showed that body condition was an important factor in the duration of the tonic immobility response; individuals with lower body condition had shorter responses independent of origin. Even though a correlation was found it is important to state that it was a weak correlation. Possibly other factors are involved in the TI responses. The results found here showed that husbandry differences, and not just being in captivity per se, had an impact on the health conditions of frogs and as a consequence affected their behavioural responses.

TI response is an acute stress response to a short term elevation of corticosterone levels, as has already been demonstrated in experiments using Fijian ground frogs (*Platymantis vitiana)* [18]. A short term elevation of stress hormones could be caused by a predator attack or the simulation of one (Tonic immobility test). A short-term increase in the corticosterone levels can promote key changes in the behaviour and physiology that enables individuals to cope with stress [19]: an acute stress response. Some of the key behaviours affected by corticosterone in amphibians are defensive behaviours such as tonic immobility [18]. However, if frogs from Mitsinjo were already experiencing chronic levels of stress due to a poor diet and environment, it is possible that their acute stress responses could be blunted [46], such as TI responses.

Body condition index can be used to assess the chronic levels of stress of captive animals [41], while TI response could be an alternative technique to asses acute stress responses on captive individuals. The stress response is not inherently detrimental, but rather, is a complex and essential negative-feedback process [47]. The capacity to cope with threatening (acute stress) situations is a vital ability to survival in the wild [35]. Predation, competition and other stressful events are part of the routine in the wild habitats.

A biosecurity facility for the conservation of amphibians on site is very important step for the future of many different species [48]. However, maintaining the necessary standards to keep animals fit for reintroductions is still a challenge. The husbandry differences, provision of UV light and diet supplementation, found between Chester Zoo and Mitsinjo reflect the availability of equipment and diet supplements in each country. Reintroductions are costly and time consuming; therefore, to make the best use of resources available it is important to screen individuals that are destined for reintroduction.

Captive environments are different from the wild and can impose different selection pressures or relaxed selection pressures leading to adaptation to captivity and, consequently, affecting behaviour including anti-predators responses [1,8,21,48]. The importance of maintaining the behavioural integrity of zoo populations, especially those that are used for conservation efforts including reintroductions is critical for the conservation of biodiversity [21]. Amphibians have long been neglected in research into animal welfare and behavioural problems related to captivity; this is clear in the historic lack of enriched captive environments to encourage natural behaviour and psychological well-being [48].
